# The Conformationally Sensitive Spatial Distance Between the TM3-4 Loop and Transmembrane Segment 7 in the Glutamate Transporter Revealed by Paired-Cysteine Mutagenesis

**DOI:** 10.3389/fcell.2021.737629

**Published:** 2021-09-21

**Authors:** Qi Qu, Ji Wang, Guiping Li, Rongqing Chen, Shaogang Qu

**Affiliations:** ^1^Department of Neurology, Nanfang Hospital, Southern Medical University, Guangzhou, China; ^2^Department of Neurobiology, School of Basic Medical Sciences, Southern Medical University, Guangzhou, China; ^3^Guangdong-Hong Kong-Macao Greater Bay Area Center for Brain Science and Brain-Inspired Intelligence, Guangzhou, China; ^4^Key Laboratory of Mental Health of the Ministry of Education, Southern Medical University, Guangzhou, China; ^5^Department of Nuclear Medicine, Nanfang Hospital, Southern Medical University, Guangzhou, China

**Keywords:** EAAT2, TM3-TM4 loop, TM7, cross-linking, paired-cysteine mutagenesis

## Abstract

Excitatory amino acid transporters can maintain extracellular glutamate concentrations lower than neurotoxic levels by transferring neurotransmitters from the synaptic cleft into surrounding glial cells and neurons. Previous work regarding the structural studies of Glt_*Ph*_, Glt_*TK*_, excitatory amino acid transporter 1 (EAAT1), EAAT3 and alanine serine cysteine transporter 2 described the transport mechanism of the glutamate transporter in depth. However, much remains unknown about the role of the loop between transmembrane segment 3 and 4 during transport. To probe the function of this loop in the transport cycle, we engineered a pair of cysteine residues between the TM3-TM4 loop and TM7 in cysteine-less EAAT2. Here, we show that the oxidative cross-linking reagent CuPh inhibits transport activity of the paired mutant L149C/M414C, whereas DTT inhibits the effect of CuPh on transport activity of L149C/M414C. Additionally, we show that the effect of cross-linking in the mutant is due to the formation of the disulfide bond within the molecules of EAAT2. Further, L-glutamate or KCl protect, and D,L-threo-β-benzyloxy-aspartate (TBOA) increases, CuPh-induced inhibition in the L149C/M414 mutant, suggesting that the L149C and M414C cysteines are closer or farther away in the outward- or inward-facing conformations, respectively. Together, our findings provide evidence that the distance between TM3-TM4 loop and TM7 alter when substrates are transported.

## Introduction

Glutamate is the predominant excitatory neurotransmitter in the brain (Curtis and Johnston, 1974; Fonnum, 1984) and is critical to numerous central nervous system (CNS) functions (Tang et al., 1999; Matsuzaki et al., 2004; Chantranupong and Sabatini, 2018; Boender et al., 2020; Stephenson-Jones et al., 2020). Anomalously elevated glutamate in the synapse results in CNS neurotoxicity; removal of glutamate occurs via glutamate transporters (excitatory amino acid transporters; EAATs), as enzymes that metabolize glutamate in the synaptic cleft are scarce. Glutamate transporters transfer the neurotransmitters over a transmembrane glutamate gradient by harnessing preexisting ion electrochemical gradients (Danbolt, 2001). These secondary active transporters belong to the solute carrier family 1 and are mainly expressed in neurons and glial cells. Among five subtypes of EAATs (EAAT1-5), EAAT2 plays an important role in catalyzing the metabolism of this excitatory transmitter. The EAAT2 subtype is expressed predominately in astroglia and is responsible for nearly 90% of glutamate transport in the forebrain (Tanaka et al., 1997; Vandenberg and Ryan, 2013; Martinez-Lozada et al., 2016). Researchers have reported that genetic mutations cause changes in the properties of important amino acid residues, leading to abnormalities in the structure and function of the transporter (Brocke et al., 2002; Zhang and Qu, 2011; Rong et al., 2015, 2016). Also, dysregulation of EAAT2 has been implicated in some neurodegenerative diseases including but not limited to amyotrophic lateral sclerosis (Takahashi et al., 2015; Jiang et al., 2019; Malik and Willnow, 2019), Alzheimer’s disease (Takahashi et al., 2015; Ugbode et al., 2017; Malik and Willnow, 2019; Sharma et al., 2019), and epilepsy (Takahashi et al., 2015; Guella et al., 2017; Malik and Willnow, 2019). For this reason, functional studies of EAAT2 are critical and may reveal potential genetically based therapeutic targets.

In addition to transporting glutamate, mammalian EAATs cotransport 3 sodium cations and 1 proton, and antiport 1 potassium cation (Zerangue and Kavanaugh, 1996; Levy et al., 1998). Indeed, when glutamate molecules are transported, an electrogenic net current with two positive charges is generated. This current depends on chloride anion conductance (activated by substrate transport and thermodynamically uncoupled) to curb the accumulation of positive charges and maintain continuous transfer (Billups et al., 1996; Ryan and Mindell, 2007).

In 2004, researchers identified and described the crystal structure of Glt_*Ph*_, a prokaryotic homolog of EAATs that originated from archaeal ortholog *Pyrococcus horikoshii* (Yernool et al., 2004). This homolog shares ∼37% sequence identity with human EAAT2 although is more conservative in the C-terminal (critical for facilitating the transport core to bind substrates and cations; Matin et al., 2020). Notably, Glt_*Ph*_ is a homotrimer, and each subunit with an independent permeation pathway has eight transmembrane segments (TM1-8) and two re-entrant hairpin loops (1 and 2; HP1-2; Yernool et al., 2004). The carboxy terminus of Glt_*Ph*_ (particularly TM7-8 and HP1-2) is actively involved in substrate transport (Yernool et al., 2004; Matin et al., 2020). Furthermore, the amino-terminal “cylinder” formed by TM1-6 secures these functional segments. Hence, each monomer “owns” an independent translocation pathway (Yernool et al., 2004), which corroborates the functions of its mammalian counterparts: a trimer with three identical and independent monomers (Gendreau et al., 2004; Koch and Larsson, 2005; Leary et al., 2007). By coupling with the ion gradient, the transporter catalyzes the movement of the substrate through the lipid bilayer, thereby facilitating the completion of the substrate transport (Gouaux, 2009). In 2017, researchers published the crystal structure of EAAT1 in both a substrate binding state and an outward facing state. This research highlighted the organization of diverse functional domains in transporters: each protomer consists of a scaffold domain containing TM1, 2, 4, and 5 as well as a conserved transport domain containing TM3, 6, 7, 8, HP1 and HP2 (Canul-Tec et al., 2017).

Substrate binding and important conformational changes are also related to the opening and closing of the gate, namely the non-helical tip of HP2 of the transporter (Garaeva et al., 2019; Wang and Boudker, 2020). Indeed, the binding of substrates and ions outside the cell is related to the opening of the tip of HP2. Movement of the tip of HP2 also allows the substrate and ions to dissociate from the inward-facing state. In the outward-facing state, when the tip of HP2 opens to expose the substrate and ion-binding sites to the extracellular solution, HP1 remains fixed. This also applies to the inward facing state where the sole gate HP2 becomes open and HP1 is locked, accompanied by the movement of the rest of the transport domain (Garaeva et al., 2019; Arkhipova et al., 2020; Wang and Boudker, 2020; Qiu et al., 2021).

**FIGURE 1:**
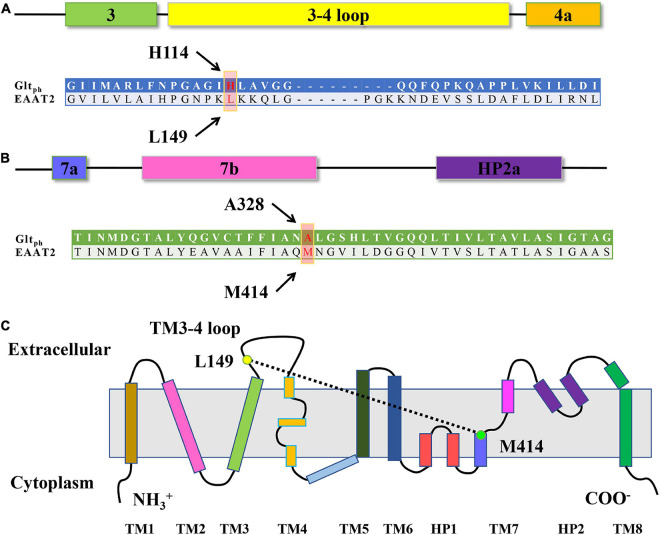
Sequence alignment and EAAT transmembrane topology. **(A,B)** Sequence alignment of the TM3-TM4 loop **(A)** and TM7 **(B)** of Glt_*Ph*_ and EAAT2. The arrows show the residues L149 and M414 of EAAT2, which occupy the positions homologous to H114 and A328 in Glt_*Ph*_. **(C)** Schematic of EAAT transmembrane topology. Yellows dots show the approximate locations of the residue L149 and M414 of EAAT2.

The loop between TM3 and TM4 undergoes multiple conformational changes during substrate transport, as confirmed by a protease cleavage experiment in 2010 (Compton et al., 2010). The crystal structure of Glt_*Ph*_ makes apparent that this long loop is situated in close proximity to transport domains. Some researchers have even shown that the TM3-4 loop rich in proline undergoes substrate-dependent conformational changes, as confirmed by limited trypsin hydrolysis analysis and fluorescein 5-maleimide accessibility experiments (Figure 1A; Compton et al., 2010). In addition, although the protein maintains its structural integrity, cleavage of the protein backbone within the TM3-4 loop through the engineered factor X protease recognition site leads to an almost complete loss of transport activity (Compton et al., 2010; Mulligan and Mindell, 2013). These findings indicate that the TM3-4 loop plays a vital role in the transportation cycle. Studies have also performed cysteine-scanning mutations on the TM3-4 loop and identified many single amino acid substitutions that cause significant changes in the activity of the transporter (Compton et al., 2010). In the prokaryotic crystal structure, for example, the TM3-4 loop undergoes a substrate-sensitive configuration change during transportation that is an important part of the transportation mechanism (Compton et al., 2010; Mulligan and Mindell, 2013). However, there is only about 12.5% sequence homology in TM3-4 loop between the eukaryotic EAAT2 and the prokaryotic Glt_*Ph*_; the conformation of EAATs TM 3–4 loop and its biological functions have not yet been fully elucidated. Studies on the crystal structure of prokaryotes Glt_*Ph*_ indicate that HP1, HP2, TM7, and TM8 may form a substrate-binding pocket during the transportation process (Yernool et al., 2004; Boudker et al., 2007; Reyes et al., 2009). In particular, when the transporter is in an inward-facing conformation, the substrate-binding region (including TM7) moves 18 Å toward the cytoplasm (Qu and Kanner, 2008; Reyes et al., 2009; Zhang et al., 2019), which indicates that TM7 has a conformation-dependent spatial position for transport and actively maintains transport functions (Figure 1B). In the eukaryotic EAAT1 heat-resistant mutant EAAT1_*cryst*_, the equivalent region in alanine-serine-cysteine transporter 2 (ASCT2) replaced TM3-4c of EAAT1 to obtain a stable crystalline EAAT1 construct. To improve crystallinity, two predicted *N*-glycosylation sites of the transporter, N155T and N204T, were introduced through specific mutations. Therefore, the undefined regions in the crystal structure include the TM3-TM4a (residues 153–173) and TM4b-c (residues 200–208) loops and the N (residues 1–28) and C (residues 490–522) termini. Because of the partial region replacement by ASCT2, the identity of TM3-4 loop between the EAAT1_*cryst*_ and the wild-type EAAT1 sequence is 16.1% (Canul-Tec et al., 2017), and the spatial relationship between TM3-4 loop residues in EAAT2 and TM7 is still unknown.

Here, we aimed to reveal the conformational change of brain transporter EAAT2 by way of transport assay on paired cysteine mutant L149C/M414C. After engineering multiple paired cysteine mutants between the TM3-4 loop and TM7, we found that oxidation severely inhibits the transport activity of L149C/M414C (Figure 1C). In oxidative conditions, when pretreated with different mediums, the transport activity of L149C/M414C varies because of the change in the distance between the two residues. Indeed, we confirmed that the residues at positions L149 and M414 in the single mutant have relatively high accessibility. These results indicate that during the transport cycle of EAAT2, distance between two residues on the TM3-4 loop and TM7 physically changed. This finding may provide evidence of the spatial structural change between these two key segments when EAAT2 ferries cargo.

## Results

### Cysteine Cross-Linking at Amino Acids L149C/M414C of CL-EAAT2 Reduces Its Transport Activity

**FIGURE 2:**
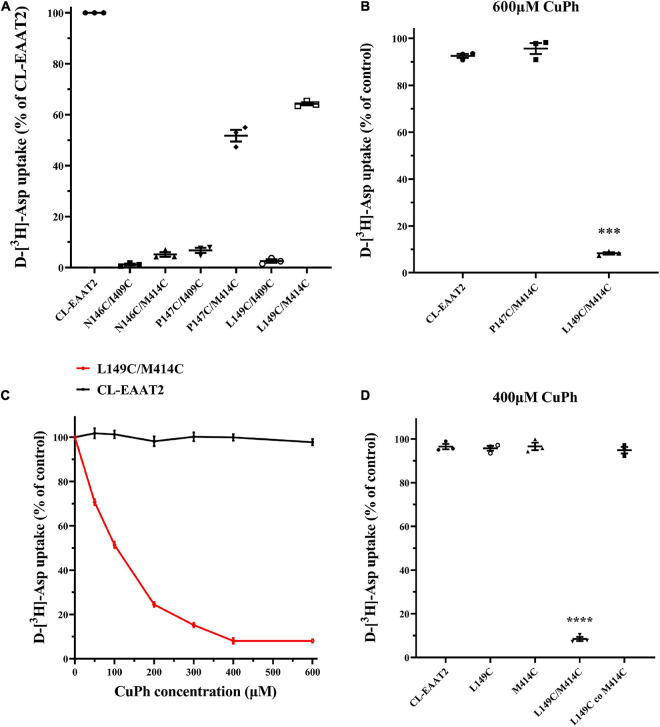
Inhibition to D-[^3^H]-Aspartate uptake of paired mutant L149C/M414C induced by CuPh. **(A)** Six paired residues on the TM3-TM4 loop and TM7 were replaced with cysteines. HeLa cells expressing the engineered cysteine mutants and cysless-EAAT2 (CL-EAAT2) were washed with choline chloride solution twice and incubated with radioactive labeled aspartate for 10 min. Transport activity of CL-EAAT2 and 6 various paired cysteine mutants were determined through the way of measuring D-[^3^H]-aspartate uptake. **(B)** D-[^3^H]-aspartate uptake was determined in CL-EAAT2, P147C/M414C and L149C/M414C with a pre-incubation of 600 μm CuPh for 5 min followed by treatment with D-[^3^H]-aspartate for 10 min. **(C)** D-[^3^H]-aspartate uptake was determined in CL-EAAT2 and L149C/M414C pre-incubated in incremental concentrations of CuPh. **(D)** D-[^3^H]-aspartate uptake was determined in CL-EAAT2, single cysteine mutants (L149C or M414C), and L149C/M414C paired cysteine mutant, and a co-transfection of L149C and M414C single mutants pre-incubated with 400 μM CuPh. Transport activities upon incubation with CuPh are normalized separately for uptake in the absence of CuPh, apart from Figure **(A)**, which is normalized to activity of CL-EAAT2. Data are the means ± S.E. from at least three separate experiments in triplicate. Values significantly different from those of CL-EAAT2 were determined by one-way ANOVA (***p < 0.001; ****p < 0.0001; *n* = 3).

To probe whether residues exist at EAAT2 TM3-4 and TM7 that are close enough to form disulfide bonds, we identified seven pairs of residues to study based on the crystal structural model of Glt_*Ph*_.; corresponding double mutations were then designed for each. We expressed these mutants in HeLa cells and selected two double mutants with relatively high transport activity: P147C/M414C and L149C/M414C (Figure 2A). The activities of the five other double mutants were less than 20% compared to the activity of CL-EAAT2, and we speculate that they may exhibit fewer transport activities in the presence of Cu(II)(1,10-phenanthroline)_3_ (CuPh), which is unfavorable under oxidative conditions. Therefore, these mutations were not used for any further experiments. However, after pretreatment with CuPh (600 μM; an oxidizing reagent that accelerates the formation of disulfide bonds between two thiol groups), we found that the activity of L149C/M414C was markedly reduced compared to CL-EAAT2, which may reflect the formation of disulfide bonds between these two residues (Figure 2B). To detect the concentration-dependence of L149C/M414C, we determined its transport activity on incremental concentrations of CuPh. When the concentration of CuPh reached to 400 μM, 91.96 ± 1.3% of L149C/M414C transport was inhibited; the half-maximal effect was close to 100 μM (51.45 ± 1.39%; Figure 2C). Thus, we used these two concentrations in subsequent experiments. Finally, we hypothesized that transport inhibition elicited by disulfide bond occurs within EAAT2 molecules, rather than between molecules. Our results are consistent with this idea, as co-transfection of L149C and M414C results in continued transport by the mutants in 400 μM CuPh conditions (Figure 2D).

### Mutants Are Deficient in Surface Expression and Transport

To probe the effect of the cysteine mutation itself on transport activity of EAAT2, we measured the activity of the indicated mutants in the absence of CuPh. The activities of L149C, M414C, and L149C/M414C decreased to 70–90% relative to that of CL-EAAT2 (Supplementary Figure 1A). To determine whether this decrease is a result of lower surface expression or deficient transport ability of EAAT2, we assayed the expression level of EAAT2. Notably, the average molar mass (Mw) of a monomer is lower than that of deglycosylated or glycosylated polymers (Supplementary Figure 1B; Yernool et al., 2004; Petr et al., 2015). Our data demonstrate no significant difference in total protein. However, M414C and L149C/M414C exhibit decreased expression in biotinylated proteins (membrane protein) and increased expression in non-biotinylated proteins (cytoplasmic protein) compared to CL-EAAT2. We therefore hypothesized that these mutants may be deficient in membrane targeting and transport but aggregate in cytoplasm (Supplementary Figures 1C–E). We found that the ratio of biotinylated proteins to non-biotinylated proteins in M414C and L149C/M414C reduced compared to CL-EAAT2 (Supplementary Figure 1F). Furthermore, the ratio of biotinylated proteins to D-[^3^H]-aspartate uptake in L149C/M414C was higher than that of CL-EAAT2 (Supplementary Figure 1G), illustrating that surface expression of M414C and L149C/M414C is lower and transport activity of double mutants is impaired, consistent with our hypothesis.

### Cysteine Cross-Linking at Amino Acids L149C/M414C of CL-EAAT2 Alters the Kinetic Parameters of Transporter

To verify whether mutation and cross-linking affect the kinetic properties of the transporter, particularly apparent substrate affinity, we measured Michaelis constant (*K*_*m*_) and the maximal transfer rate (*V*_*max*_) of CL-EAAT2, L149C, M414C, and L149C/M414C, with or without pre-incubation of 400 μM CuPh, respectively, (Table 1). Compared to pretreatment without CuPh, L149C/M414C with pretreatment exhibited reduced *V*_*max*_ and increased *K*_*m*_, indicating impaired transport function and decreased apparent affinity due to CuPh exposure, both of which are the result of disulfide cross-linking. However, single mutants with or without CuPh pretreatment were not significantly different, and it is possible that there was no disulfide bond formation after adding CuPh.

**TABLE 1:** Kinetic parameters of EAAT2 mutants in the absence or presence of CuPh.

	**Without CuPh**		**With CuPh**	
	***V*_*max*_ (%)**	***K*_*m*_ (μm)**	***V*_*max*_ (%)**	***K*_*m*_ (μm)**
CL-EAAT2	100	30.9 ± 9.7	106.7 ± 28.5	31.3 ± 11.0
L149C	88.6 ± 16.1	33.9 ± 8.6	82.3 ± 16.9	31.5 ± 8.7
M414C	87.5 ± 15.2	35.7 ± 8.8	84.4 ± 14.1	34.9 ± 8.0
L149C/M414C	74.2 ± 15.4	33.2 ± 9.5	12.5 ± 1.9^***^	60.9 ± 14.4^*^

D-[^3^H]-aspartate uptake was measured in HeLa cells expressing CL-EAAT2 and indicate mutants. *V*_*max*_ and *K*_*m*_ of CL-EAAT2, L149C, M414C, and L149C/M414C were derived from the fitted Hill equation, where *V*_*max*_ and *K*_*m*_ were normalized to CL-EAAT2 without CuPh, respectively. Data are the means ± S.E. representative of at least three separate experiments in triplicate. Values which were significantly different from those of CL-EAAT2 were determined by one-way ANOVA (*p < 0.05; ***p < 0.001; *n* = 3).

### Dithiothreitol Inhibits the Effects of Cross-Linking L149C/M414C on Transport Activity

**FIGURE 3:**
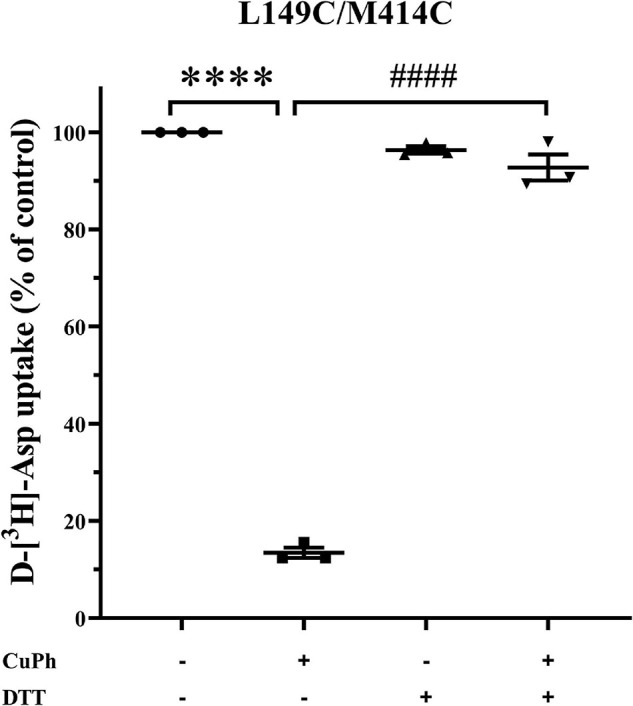
Effect of Dithiothreitol on CuPh-induced inhibition in L149C/M414C double mutants. HeLa cells expressing L149C/M414C were preincubated for 5 min in the presence and absence of 400 μM CuPh and then incubated with or without 20 mM DTT. Transport of D-[^3^H]-aspartate was then determined. Transport activity of L149C/M414C is normalized for that of CL-EAAT2 without CuPh and DTT. Data are the means ± S.E. representative of at least three separate experiments in triplicate. Values significantly different from those of double mutants with CuPh were determined by one-way ANOVA (^****^p < 0.0001; ^####^*p* < 0.0001; *n* = 3).

The experiments presented henceforth demonstrate reduced transport activity and apparent affinity of double mutants L149C/M414C induced by CuPh. However, whether CuPh triggers a disulfide bond between the two mutated cysteine residues remained unclear. Hence, HeLa cells expressing L149C/M414C were preincubated for 5 min in the presence and absence of 400 μM CuPh, followed by incubation with or without 20 mM DTT. Then, D-[^3^H]- aspartate uptake was measured. Compared to double mutants L149C/M414C treated with CuPh alone, the transport activity of double mutants treated with CuPh and DTT was restored to 91.9 ± 1.3% relative to CL-EAAT2 (Figure 3). This indicates that the reduction of disulfide bonds results in recovery of activity and supports the finding that the presence of disulfide bonds leads to reduced activity.

### Allostery of the Transporter Influences the Effect of Cross-Linking on Double-Cysteine Mutants

**FIGURE 4:**
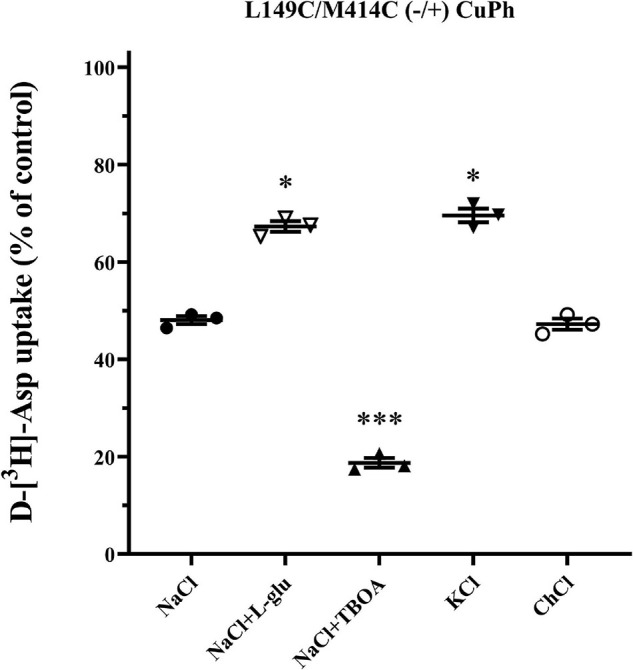
Effects of outer medium on CuPh-induced inhibition in L149C/M414C double mutants. HeLa cells expressing the indicated double mutants were treated with 100 μM CuPh mixed with different mediums (150 mM NaCl solution, NaCl solution + 1 mM L-glutamate, NaCl solution + 20 μM TBOA, 150 mM KCl solution, and 150 mM choline chloride solution). D-[^3^H]-aspartate uptake was assayed for 10 min. Values are given as a percent of control (pre-incubation without CuPh). Data are the means ± S.E. representative of at least three separate experiments in triplicate. Values significantly different from L149C/M414C treated with 100 μM CuPh in NaCl solution were determined by one-way ANOVA (^∗^*p* < 0.05; ^∗∗∗^*p* < 0.001; *n* = 3).

To investigate whether the inhibitory effect of CuPh on transport activity is conformationally dependent, we measured the transport activity of double mutants L149C/M414C in various outer mediums combined with CuPh, including L-glutamate, potassium and TBOA. By using L-glutamate and potassium, both cargo of EAAT2, the proportion of intracellular facing conformation of the transporters increases (Shlaifer and Kanner, 2007; Qu and Kanner, 2008). We also used TBOA, a non-transportable substrate analog, to raise the ratio of extracellular facing conformation (Qu and Kanner, 2008). When the proportion of outward facing state was increased, the transport activity of mutants was inhibited, which may be indicative of the proximity between two mutant residues. Furthermore, when the mutated EAAT2 shifted to the inward facing conformation, its activity was restored, which suggests that the two mutant residues are further away from each other in this conformation (Figure 4).

### Aqueous Accessibility of Single-Cysteine Mutant at L149C Changes

**FIGURE 5:**
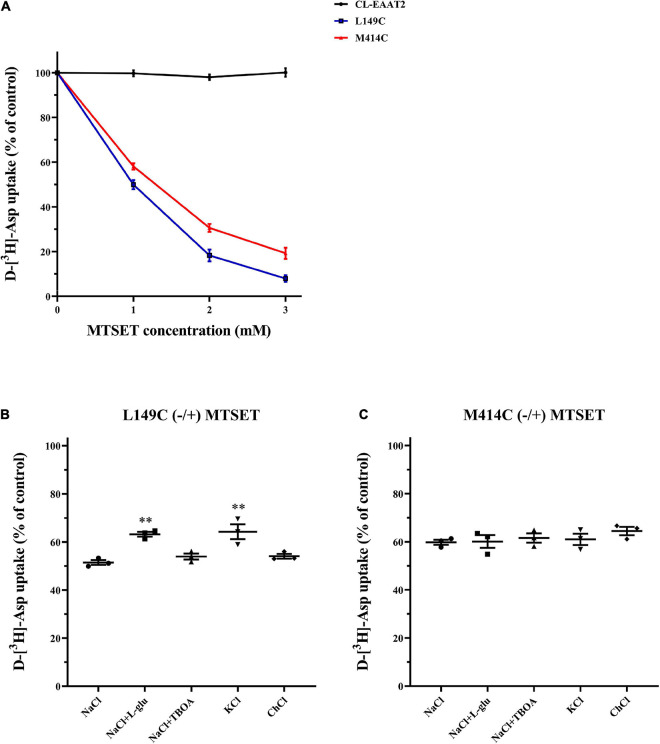
Effects of outside medium on MTSET-induced inhibition in single mutants L149C and M414C. **(A)** D-[^3^H]-aspartate uptake was determined in CL-EAAT2, L149C and M414C pre-incubated in incremental concentrations of MTSET. HeLa cells expressing L149C **(B)** and M414C **(C)** were treated with 1 mM MTSET mixed with different mediums (150 mM NaCl solution, NaCl solution + 1 mM L-glutamate, NaCl solution + 20 μM TBOA, 150 mM KCl solution, and 150 mM choline chloride solution). Transport activity of L149C or M414C was normalized to that of two single mutants treated in corresponding medium without MTSET. Data are the means ± S.E. and representative of at least three separate experiments. Values significantly different from L149C or M414C treated with 1 mM MTSET in NaCl solution were determined by one-way ANOVA (^∗∗^*p* < 0.01; *n* = 3).

Based on our finding that the distance between two mutated cysteine residues changes in different conformations, we assumed that during the transport cycle, aqueous accessibility of mutant residues is altered. Hence, in the above experiments, both distance and accessibility of extracellular CuPh may have affected our results. Thus, we used impermeable thiol reagent (2-trimethylammonium) ethylmethanethiosulfonate (MTSET), which covalently binds to cysteine and inhibits transporter activity, to examine aqueous accessibility. Notably, the half-maximal concentration of MTEST is nearly 1 mM for both single cysteine mutants and therefore suitable for the current research question (Figure 5A). Further, L-glutamate and potassium protect mutants from inhibition of MTSET in L149C, but TBOA does not (Figure 5B). Hence, the decreased accessibility of L149C in the presence of L-glutamate and potassium may partially explain why the transport activity of double mutants increased when facing inward in the presence of CuPh. However, for M414C, neither substrates nor non-transportable analogs inhibited transport activity of the single mutant (Figure 5C), suggesting that the aqueous accessibility of the mutated transporter was unaffected during substrate transfer.

## Discussion

The TM3-TM4 loop has been previously characterized by the crystal structure model, and is connected to both the anchored TM4 and the ambulatory TM3; the loop spans across critical segments for transport including HP2, TM7, and TM8 (Yernool et al., 2004; Canul-Tec et al., 2017). However, the role of this loop in the transport of glutamate and the relative relationship of the loop to these segments remain largely unknown (Compton et al., 2010; Mulligan and Mindell, 2013; Wang and Qu, 2021). Here, based on the crystal structure of three different conformations of Glt_*Ph*_, we designed the double mutant L149C/M414C located on the TM3-TM4 loop and TM7 to probe how this loop participates in the translocation and conformation change relative to TM7. In the crystal structure of substrate binding state and outward facing state of EAAT1, the introduction of substitutions or mutations necessary to complete stable crystallization results in an unresolved TM3-TM4 loop, which may disrupt the crystallization (Canul-Tec et al., 2017). Hence, we merely refer to the model of Glt_*Ph*_ for designation.

The transport activity of double mutant L149C/M414C is lower than cys-less EAAT2, although much higher than other double mutants in the absence of CuPh (Figure 2A). We demonstrate that this difference occurs because of the reduced apparent affinity or surface expression of L149C/M414C. Indeed, the kinetic experiment of L149C/M414C shows that the substrate affinity and maximal transport rate decrease following the treatment of CuPh; this decrease may be a result of the formation of disulfide bond (Table 1). However, western blot assays in L149C/M414C show impaired membrane targeting, as the mutants appear to aggregate in cytoplasm. The impaired ability of membrane targeting in double cysteine mutants may be due to its retention in the endoplasmic reticulum in an immature form. Indeed, an experiment published in 2004 reports that some conserved residues between TM7 and TM8 play a vital role in the forward trafficking of EAAT2 (Kalandadze et al., 2004). In contrast, we observed no relationship between our western blot assays and the reduced activity of L149C, likely because the accumulated effect results in reduced activity, despite no significant difference between L149C and CL-EAAT2 (Supplementary Figures 1F,G).

Here, we demonstrate that, compared to the double mutant, co-transfected single mutants are resistant to the inhibition of CuPh (Figure 2D). Hence, intramolecular cross-linking results in inhibition of transport activity. We also assayed the activity of L149C/M414C in the presence of DTT and demonstrate that pre-incubation with CuPh and subsequent treatment with DTT results in comparable activity of L149C/M414C and double mutants that were not exposed to any treatment. This activity was also significantly different from the activity of double mutants that were treated with CuPh alone (Figure 3), suggesting that incubation with CuPh results in the formation of disulfide bonds.

**FIGURE 6:**
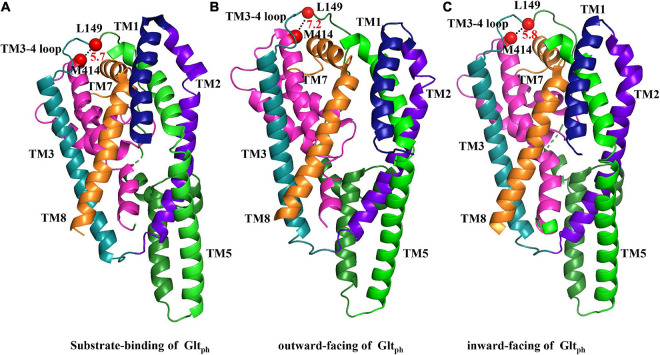
Substrate-binding and outward- and inward-facing structures of Glt_*Ph*_. The crystallized substrate-binding **(A)**, outward-facing **(B)**, and inward-facing **(C)** forms of Glt_*Ph*_ are shown (respective PDB ID codes 1XFH, 2NWW, and 2NWX) and were aligned as indicated in figure. Segments that are not labeled are TM4 (dark green), and HP2 (pink). TM6 and HP1 were removed for the facilitation of observation. The distances between the two amino acids are represented by black dashed lines between two red dots, which indicate the spatial locations of L149 and M414, respectively.

The crystal structure of bacterial homolog Glt_*Ph*_ and eukaryotic EAAT1 could aid in the exploration of the relative motion of different segments in glutamate transporters. To determine the relative conformational change between the TM3-TM4 loop and TM7 during the transport cycle, we measured the transport activity of double mutant L149C/M414C in different extracellular mediums. TBOA, a substrate analog with aspartate moiety and a bulky benzyl group, increases the extracellular facing state of the transporter (Qu and Kanner, 2008). After pre-incubation with TBOA, the activity of L149C/M414C was lower than those in NaCl solution, which represents the substrate binding state or “occluded” state (Shlaifer and Kanner, 2007; Reyes et al., 2009; Figure 4). Notably, when the transporter increased its outward ratio, these two residues became closer. Extracellular potassium ions or L-glutamate also reversed the orientation of substrate influx and increased the ratio of cytoplasm facing conformation of glutamate transporters (Shlaifer and Kanner, 2007; Qu and Kanner, 2008; Crisman et al., 2009). We found that pretreatment with potassium ions or L-glutamate protects L149C/M414C from inhibition by CuPh, as activity was restored compared with the activity of L149C/M414C in NaCl solution (Figure 4). This suggests that two residues are likely distanced from each other. We also compared our findings with the published crystal structure of Glt_*Ph*_ by measuring the Cα–Cα distance between His114 and Ala328, which are equivalent to Leu149 and Met414 in EAAT2 in models representative of different conformations of this homolog. When Glt_*Ph*_ transitioned to the inward facing state, the distance between two residues does not change substantially (Figures 6A,C). However, when Glt_*Ph*_ transitioned to the outward facing state, the distance between the two residues increased (Figures 6A,B). The distance we observed in our experiments is different from that shown by the Glt_*Ph*_ crystal structure. The reasons for this difference are as follows. First, prokaryotic Glt_*Ph*_ and eukaryotic EAATs originate from different species that are evolutionarily different (sequence homology ∼12.5% in TM3-4 ring between eukaryotic EAAT2 and prokaryotic Glt_*Ph*_), although important functional segments are highly conserved (Vandenberg and Ryan, 2013). Second, mutations were made in the published crystal structure model of Glt_*Ph*_ [i.e., seven His residues in non-conserved site of Glt_*Ph*_ (Yernool et al., 2004)] that may have caused the crystal structure models to differ from the structure of the transporters in their native membrane environment. The TM3-4 loop was not completely resolved in the structure of EAAT1 and EAAT3; however, in the Cryo-electron microscopy structure of EAAT3 in the outward facing state (PDB ID code 6 × 2Z), Lys123 (namely the Leu149 in EAAT2) was resolved, and we measured the Cα–Cα distance between this site and Leu384 (Met414 in EAAT2). The distance between the two sites is 4 Å, and this proximity might support our finding that the two residues become closer together in an outward facing state (Figure 4). Furthermore, the distance is shorter compared with its counterpart in the model of Glt_*Ph*_ in the outward facing state (Figure 6B; Canul-Tec et al., 2017; Qiu et al., 2021).

We also considered the alternation of accessibility of single mutants L149C and M414C. As mentioned, TBOA, L-glutamate and potassium ions influence the conformation of glutamate transporters. Using HeLa cells pre-incubated with MTSET, we show that application of analog TBOA does not protect the single mutant from the inhibition of MTSET in L149C, although treatment with extracellular potassium ions and L-glutamate was capable of doing so (Figure 5B). These three agents were incapable of reversing or reducing the transport activity of M414C (Figure 5C). Combined, these data suggest that the aqueous accessibility of L149C increased when EAAT2 shifted to the inward facing conformation. We also show that application of CuPh decreases the apparent affinity of L149C/M414C and we suggest that in this conformation, where two cysteine residues are in close proximity, the formation of the disulfide bond disrupts the movement of the two residues and hinders transport of the substrate. We also speculate that this may occur because M414C, located in the TM7, is proximal to several residues that participate in the sodium binding in the homolog Glt_*Ph*_ (Boudker et al., 2007; Qiu et al., 2021). Furthermore, the residues at positions L149 and M414 in the single mutant have relatively high accessibility, and we suggest that the TM3-TM4 loop may be supported by the L149C to function and ultimately aids in substrate translocation.

## Conclusion

Our findings indicate that when EAAT2 ferries its cargo, the TM3-TM4 loop undergoes a proximal conformational change to TM7. Here, we report the significant role of the TM3-4 loop in the transport cycle and outline the interaction with key transport segment TM7.

## Materials and Methods

### Cell Growth and Expression of EAAT2

HeLa cells were cultured in Dulbecco’s modified Eagle’s medium (Invitrogen, Carlsbad, CA, United States) containing 8% fetal bovine serum (Invitrogen), 200 U/mL penicillin (Beyotime Biotechnology, Shanghai, China) and 200 mg/mL streptomycin (Beyotime Biotechnology; Wang and Qu, 2021). Plasmids encoding CL-EAAT2, L149C, M414C, and L149C/M414C were transfected (Qu et al., 2019; Wang and Qu, 2021) and two single mutant constructs were co-transfected into HeLa cells infected with recombinant vaccinia/T7virus vTF7-3 (Fuerst et al., 1986) and Lipo6000 Transfection Reagent (Beyotime Biotechnology). Procedures to determine the uptake of D-[^3^H]-aspartate (PerkinElmer, MA, United States; 0.4 μCi/0.15 μM) were conducted as previously described (Qu et al., 2019; Wang and Qu, 2021) and data presented here removed the values of HeLa cells expressing the vector pBluescript SK(−) alone (Grunewald et al., 2002).

### Transport Assays

For the D-[^3^H]-aspartate uptake assay, HeLa cells expressing mutant or wild-type EAAT2 were seeded into a 24-well plate, and the uptake experiment was performed the next day. Cells were washed twice with 1 mL choline chloride (ChCl) solution, then mixed with 200 μL NaCl solution and 0.4 μCi (0.15 μM). D-[^3^H]- aspartate was then added to the cells and incubated for 10 min at room temperature. Cells were then rinsed twice with ice-cold sodium solution. Finally, they were lysed with 1% sodium dodecyl sulfate, and the accumulation of radiolabeled substrate in HeLa cells was determined by liquid scintillation counting.

### Effects of Substrate and Inhibitors on Sulfhydryl Modification and Inhibition of CuPh

Procedures used here were recently described (Qu et al., 2019; Wang and Qu, 2021). Briefly, cells in 24-well plates were washed twice with 1 mL 150 mM ChCl solution, followed by CuPh or MTSET (Invitrogen) exposure with an incubation for 5 min. Then the cells were washed twice with 1 mL of ChCl solution followed by the transport assay using 200 μL of NaCl-containing 0.4 μCi of D-[^3^H]-aspartate for 10 min. After the incubation of the radiolabeled substrate, the medium was removed, and all cells were washed twice with 1 mL of ice-cold NaCl solution, and then utilized for the transport test. We performed a preliminary titration experiment to determine the optimal CuPh and MTSET concentration for the specified mutant. The following reagents together with MTSET or CuPh were added to the pre-culture medium for inhibition assay: 150 mM NaCl solution, 150 mM NaCl solution + 1 mM L-glutamate, 150 mM NaCl solution + 20 μM DL-TBOA (Tocris, Bristol, United Kingdom), 150 mM KCl solution or 150 mM ChCl solution; subsequent transport tests were carried out.

### Dithiothreitol-Induced Reduction and Kinetic Assays

Briefly, HeLa cells were washed twice with 1 mL ChCl solution. They were then incubated with 200 μL CuPh for 5 min, and repeatedly washed with ChCl solution. HeLa cells were treated with 200 μL DTT (Sigma, St Louis, MO, United States) and washed with ChCl solution. D-[^3^H]-aspartate uptake was assayed for 10 min. In the kinetic assay, different concentrations of aspartate containing the same dose of D-[^3^H]-aspartate were applied to the HeLa cells for 10 min with or without pre-incubation with CuPh. The concentration gradients of aspartate were 1, 30, 50, 100, 200, 400, and 800 μM, and the proportions of radiolabeled/non-radiolabeled substrate were 1:6, 1:195, 1:325, 1:650, 1:1300, 1:2600, and 1:5200, respectively. *V*_*max*_ is expressed as a percentage of the *V*_*max*_ of CL-EAAT2 untreated with CuPh. *K*_*m*_ and *V*_*max*_ were measured with non-linear fitting to the generalized Hill equation using Origin 7.5 software (Northampton, MA, United States).

### Statistical Analysis

Data are presented as mean ± standard error (SE) from three independent experiments. Student’s *t*-tests (for comparison between two groups) or one-way analyses of variance (for comparison of more than two groups) were used for statistical analyses via SPSS 21.0 statistical software. Differences were considered statistically significant at *p* < 0.05 (^∗^*p* < 0.05, ^∗∗^*p* < 0.01, ^∗∗∗^*p* < 0.001, and ^****^p < 0.0001).

## Data Availability Statement

The original contributions presented in the study are included in the article/Supplementary Material; further inquiries can be directed to the corresponding author/s.

## Author Contributions

SQ and RC designed the research. QQ and JW performed the experiments. SQ, QQ, JW, and GL analyzed the data. SQ, QQ, and JW wrote the manuscript. All authors: finalization and approval of the content of the manuscript.

## Conflict of Interest

The authors declare that the research was conducted in the absence of any commercial or financial relationships that could be construed as a potential conflict of interest.

## Publisher’s Note

All claims expressed in this article are solely those of the authors and do not necessarily represent those of their affiliated organizations, or those of the publisher, the editors and the reviewers. Any product that may be evaluated in this article, or claim that may be made by its manufacturer, is not guaranteed or endorsed by the publisher.

## Abbreviations

CL-EAAT2: cysteine-less EAAT2
CuPh: Cu(II)(1,10- phenanthroline)_3_
DL-TBOA: D,L-threo-β-benzyloxy-aspartate
DTT: DL-dithiothreitol
EAATs: excitatory amino acid transporters
Glt_*Ph*_: aspartate transporter from *Pyrococcus horikoshii*
HP: hairpin loop
MTSET: (2-trimethylammonium) ethylmethanethiosulfonate
TM: transmembrane domain.
