# Supplementary material for: The Conformationally Sensitive Spatial Distance Between the TM3-4 Loop and Transmembrane Segment 7 in the Glutamate Transporter Revealed by Paired-Cysteine Mutagenesis
Source: Front Cell Dev Biol. 2021 Sep 21;9:737629. doi: 10.3389/fcell.2021.737629 (PMC8490817; doi:10.3389/fcell.2021.737629)
Supplement: Supplementary file 1 [file Data_Sheet_1.docx]

**Supplement Material**


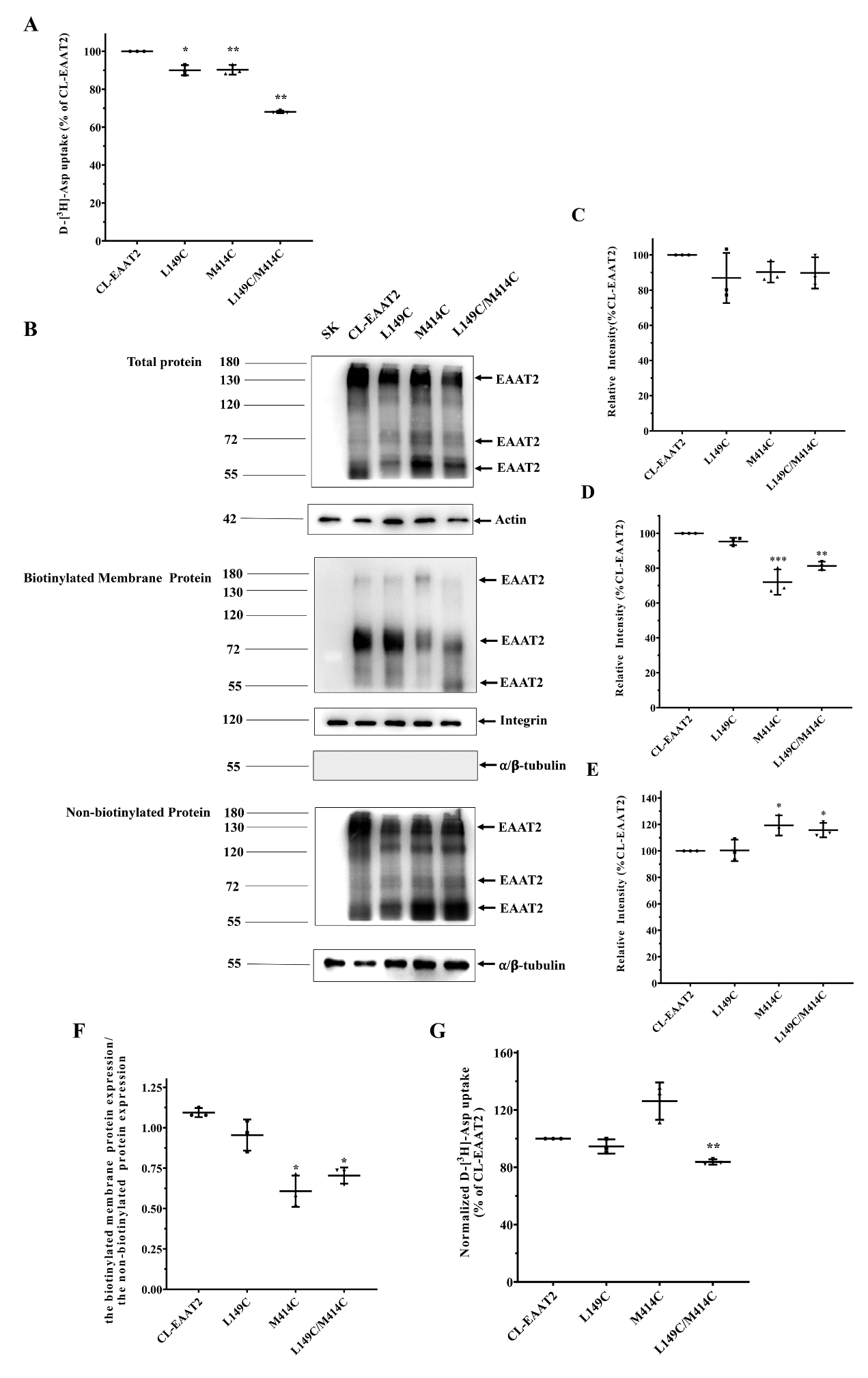


**Supplementary Figure 1. Differential protein expression of CL-EAAT2 and mutant transporter.** HeLa cells expressing single cysteine mutants, double cysteine mutants, CL-EAAT2 or an empty vector were treated and EAATs were solubilized and purified as described in “Methods”. (A) The transport activity of CL-EAAT2 and the indicated mutants were determined by measuring D-[^3^H]-aspartate uptake. (B) SDS-PAGE analysis of the expression of total, biotinylated, non-biotinylated proteins, and EAAT were visualized via western blotting techniques. Actin, internal plasma membrane marker-integrin and endogenous cytosolic protein α/β-tubulin were assessed as loading controls for the total, biotinylated, non-biotinylated proteins, respectively. α/β-tubulin was assessed to indicate whether biotinylated proteins separated completely from non-biotinylated proteins. (C-E) Quantitative analysis of protein-band intensity for total proteins, normalized to β-actin. (C) Biotinylated proteins normalized to integrin (D) and non-biotinylated proteins normalized to α/β-tubulin (E). Values are normalized to the intensity of CL-EAAT2. (F) Ratio of biotinylated proteins to non-biotinylated proteins in HeLa cells expressing CL-EAAT2 and indicated mutants. (G) Ratio of biotinylated proteins to D-[^3^H]-aspartate uptake in CL-EAAT2 and indicated mutants. Values are normalized to the intensity of CL-EAAT2. Data are the means ± S.E. representative of at least three separate experiments in triplicate. Values significantly different from those of CL-EAAT2 were determined by one-way ANOVA. (**p* < 0.05; ***p* < 0.01; ****p* < 0.001; *n* = 3).

**METHODS**

**Cell Surface Biotinylation and Western Blotting**. HeLa cells were plated onto 6-well plates, and after 24 hours, they were transfected with plasmid encoding L149C, M414C, L149C/M414C and CL-EAAT2. After transfection, HeLa cells were washed with cold PBS and incubated with 0.5 mg/mL EZ-Link Sulfo-NHS-SS-Biotin (Thermo Scientific, Waltham, MA, USA) in PBS (pH 8.0) for 20 minutes. The treated cells were then washed twice with PBS containing 100 mM glycine (Sangon biotech, Shanghai, China) to remove unreacted biotinylation reagent. Cell cultures were lysed in cell lysis buffer on ice for 20 minutes. Cell debris was removed by centrifugation at 12000 *g* at 4°C for 20 minutes. After centrifugation, supernatant was transferred to a new tube and combined with 200 μL streptavidin agarose beads (Thermo Scientific, Waltham, MA, USA) to separate cell membrane proteins. The protein samples then were collected by centrifugation (total protein, biotinylated protein, and non-biotinylated protein). Once collected, biotinylated and non-biotinylated proteins were assayed using Western blotting techniques. First, samples were boiled in the loading buffer and separated from each other though 10% SDS-PAGE. They were then transferred onto polyvinylidene difluoride membranes. After blockage with 5% bovine serum albumin (Beyotime Biotechnology), membranes were probed by the anti-EAAT2 antibody (Abcam, Cambridge, UK). Actin (Proteintech, WuHan, China), integrin (CST, Danvers, MA, USA) and α/β-tubulin (Beyotime Biotechnology) were used as internal controls for total protein, biotinylated protein, and non-biotinylated protein respectively. Bands were visualized using a Tanon 4600SF (Tanon, Shanghai, China) and data from the Western blot were analyzed using ImageJ. Values were normalized to the internal reference protein.
